# Can p63 serve as a biomarker for giant cell tumor of bone? A Moroccan experience

**DOI:** 10.1186/1746-1596-7-130

**Published:** 2012-09-27

**Authors:** Nawal Hammas, Chbani Laila, Alaoui Lamrani My Youssef, El Fatemi Hind, Taoufiq Harmouch, Tizniti Siham, Amarti Afaf

**Affiliations:** 1 Department of Pathology, HASSAN II University Hospital, Km 2.200 Route de Sidi. Harazem, Fez, 30000, Morocco; 2 Department of Radiology, HASSAN II University Hospital, Km 2.200 Route de Sidi. Harazem, Fez, 30000, Morocco

**Keywords:** P63, Bone, Giant cell tumor, Immunohistochemistry

## Abstract

**Background:**

Multinucleated giant cell-containing tumors and pseudotumors of bone represent a heterogeneous group of benign and malignant lesions. Differential diagnosis can be challenging, particularly in instances of limited sampling. The purpose of this study was to evaluate the contribution of the P63 in the positive and differential diagnosis of giant cell tumor of bone.

**Methods:**

This study includes 48 giant cell-containing tumors and pseudotumors of bone. P63 expression was evaluated by immunohistochemistry. Data analysis was performed using Epi-info software and SPSS software package (version 17).

**Results:**

Immunohistochemical analysis showed a P63 nuclear expression in all giant cell tumors of bone, in 50% of osteoid osteomas, 40% of aneurysmal bone cysts, 37.5% of osteoblastomas, 33.3% of chondromyxoide fibromas, 25% of non ossifiant fibromas and 8.3% of osteosarcomas. Only one case of chondroblastoma was included in this series and expressed p63. No P63 immunoreactivity was detected in any of the cases of central giant cell granulomas or langerhans cells histiocytosis. The sensitivity and negative predictive value (NPV) of P63 immunohistochemistry for the diagnosis of giant cell tumor of bone were 100%. The specificity and positive predictive value (PPV) were 74.42% and 59.26% respectively.

**Conclusions:**

This study found not only that GCTOB expresses the P63 but it also shows that this protein may serve as a biomarker for the differential diagnosis between two morphologically similar lesions particularly in instances of limited sampling. Indeed, P63 expression seems to differentiate between giant cell tumor of bone and central giant cell granuloma since the latter does not express P63. Other benign and malignant giant cell-containing lesions express P63, decreasing its specificity as a diagnostic marker, but a strong staining was seen, except a case of chondroblastoma, only in giant cell tumor of bone. Clinical and radiological confrontation remains essential for an accurate diagnosis.

**Virtual slides:**

The virtual slide(s) for this article can be found here: http://www.diagnosticpathology.diagnomx.eu/vs/1838562590777252.

## Introduction

Giant cell tumour of bone (GCTOB) is the prototype of giant cell rich neoplasms of the skeleton. The term giant cell tumour was coined by Bloodgood in 1912 [1] and it was not until 1940 that Jaffe distinguished giant cell tumour of bone from other bone tumours containing many osteoclast-like giant cells [2]. This lesion represents 4% to 5% of all primary bone tumors and mainly occurs in skeletally mature patients (peak incidence between ages 20 and 45 years) with a slight female predominance [3-5]. It most commonly arises at the epiphyses of long bones like the distal femur, proximal tibia, distal radius and proximal humerus [6]. This tumor can be locally aggressive with a tendency for recurrence. Lung metastases occur infrequently; more rarely, this tumor behaves as a sarcoma [4,7]. Because of its different evolution and prognosis, GCTOB must be distinguished from other multinucleated giant cell-containing tumors and pseudotumors. Differential diagnosis can be challenging, particularly in instances of limited sampling such as with needle-core biopsies. It is based not only on histology, but also on clinical and radiological data. There is currently no well-accepted diagnosis marker available for GCTOB, but recent studies using immunohistochemistry and molecular methods have demonstrated overexpression of p63 in the stromal cells of most giant cell tumors of bone and advocate its use as a diagnostic marker [3,4,6]. P63 was identified in 1998 [8]. It belongs to the family of transcription factors that also includes p53 and p73 [9]. It is mostly used as a diagnostic aid in breast, prostate, and salivary gland cancer because of its high sensitivity and specificity for mammary and salivary myoepithelial cells and prostatic basal cells [3,10-12]. It can be a useful tool in distinguishing urothélial carcinoma from prostatic carcinoma [13] and it can also be used as a prognosis factor as in adenoid cystic carcinoma [14].

The purpose of this study is to determine whether GCTOB expresses p63, and whether p63 can be used as a biomarker to discriminate GCTOB from other giant cell-rich tumors.

## Methods

**Figure 1:**
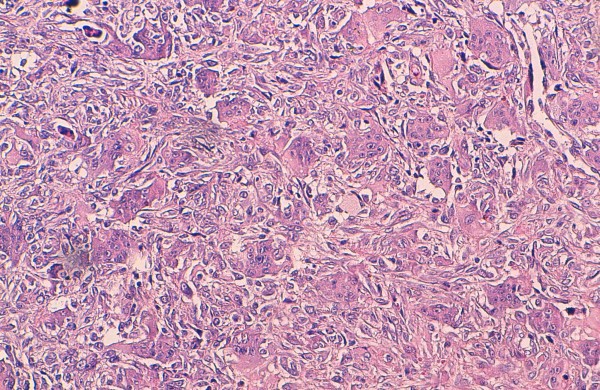
**Histological findings of giant cell tumor of bone: the tumor is composed of round mononuclear stromal cells and uniformly scattered multinucleated giant cells, many of which contain a large number of nuclei.** Characteristically, the nuclei of both stromal and giant cells are very similar. (hematoxylin-eosin stain, original magnification × 200).

**Figure 2:**
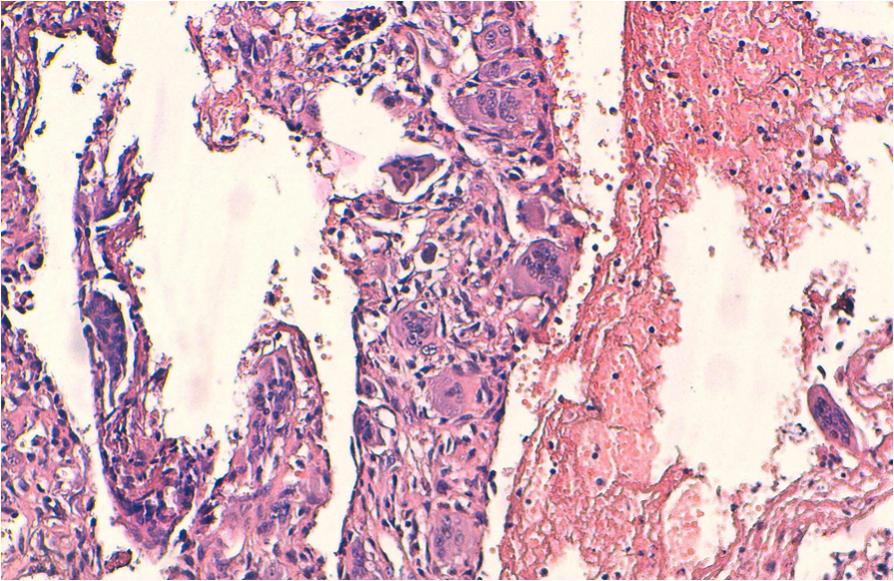
Histological findings of aneurysmal bone cyst: the tumor is composed of blood-filled cystic spaces lined by fibrous septa that are composed of uniform fibroblasts and multinucleated giant cells (hematoxylin-eosin stain, original magnification × 200).

**Figure 3:**
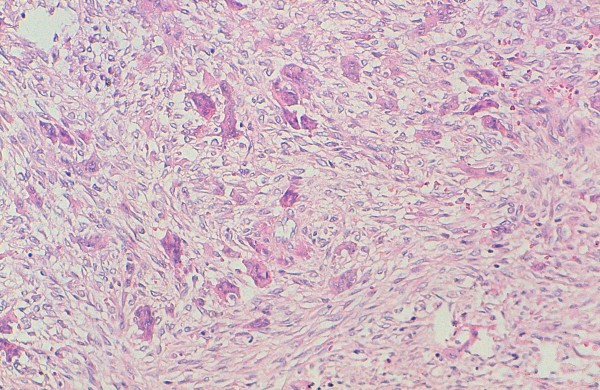
**Histological findings of central giant cell granuloma: the tumor consists of spindled fibroblasts admixed with numerous multinucleated giant cells that tend to be arranged in small clusters.** They contain fewer nuclei than seen in giant cell tumour of bone. Scattered lymphocytes are present (hematoxylin-eosin stain, original magnification × 200).

This study concerns 48 giant cell-containing tumors and pseudotumors of bone that were retrieved from department of pathology of Hassan II University Hospital in Fez, from January 2009 to February 2012. They include 12 osteosarcomas, 8 osteoblastomas, 5 GCTOB (Figure 1), 5 aneurysmal bone cysts (ABCs) (Figure 2), 4 osteoid osteomas (OO), 4 central giant cell granulomas (CGCGs) (Figure 3), 4 non ossifiant fibromas (NOFs), 3 chondromyxoid fibromas (CMFs), 1 fibrous dysplasia (FD), 1 chondroblastoma and 1 Langerhans cell histiocytosis (LCH). The data were collected prospectively from pathology reports, from forms filled by trauma surgeons, pediatric surgeons and otorhinolaryngologists, and from radiographs. A form was filled for each patient, including the following informations: patient’s name, age, sex, tumor location, histological type and P63 expression. The demographic data and location of these cases are shown in Table 1.

**Table 1:** Demographic data and location of tumours

**Case number**	**Diagnosis**	**Age**	**Sex**	**Location**
1	*osteosarcoma*	34	F	femur
2	*osteosarcoma*	40	F	femur
3	*osteosarcoma*	19	M	femur
4	*osteosarcoma*	19	M	femur
5	*osteosarcoma*	12	F	femur
6	*osteosarcoma*	14	F	femur
7	*osteosarcoma*	23	M	humerus
8	*osteosarcoma*	21	F	humerus
9	*osteosarcoma*	20	M	humerus
10	*osteosarcoma*	25	M	Tibia
11	*osteosarcoma*	18	F	Tibia
12	*osteosarcoma*	27	M	mandible
13	osteoblastoma	14	M	femur
14	osteoblastoma	19	F	femur
15	osteoblastoma	20	F	radius
16	osteoblastoma	25	F	Cuneiform bone
17	osteoblastoma	23	M	5th metatarsal bone
18	osteoblastoma	23	M	astragal
19	osteoblastoma	21	M	mandible
20	osteoblastoma	57	M	vertebrae
21	GCTOB	29	F	femur
22	GCTOB	30	M	femur
23	GCTOB	21	F	5th metacarpal bone
24	GCTOB	21	F	Fibula
25	GCTOB	40	F	Tibia
26	ABC	19	F	Fibula
27	ABC	14	F	Fibula
28	ABC	16	M	Femur
29	ABC	13	F	Tibia
30	ABC	15	M	1^st^ metatarsal bone
31	OO	30	F	femur
32	OO	28	M	femur
33	OO	24	M	astragal
34	OO	24	M	Fibula
35	CGCG	9	M	mandible
36	CGCG	15	M	mandible
37	CGCG	48	F	Maxilla
38	CGCG	53	F	Maxilla
39	NOF	16	F	Tibia
40	NOF	19	M	Tibia
41	NOF	16	F	Tibia
42	NOF	8	M	femur
43	CMF	23	M	Toe
44	CMF	33	M	Tibia
45	CMF	59	F	Sphenoid bone
46	FD	19	M	Femur
47	chondroblastoma	23	M	calcaneus
48	LCH	7	M	Ilium

M: male; F: female.

All specimens were fixed in 10% buffered formalin, embedded in paraffin and 4 micron-thick sections were stained with hematoxylin and eosin for routine histological examination.

### Immunohistochemical staining

P63 expression was evaluated by immunohistochemistry. All immunohistochemical stains were performed on a Ventana Benchmark LT automated immunostainer, on 3 micron-thick sections that were incubated with a mouse monoclonal antibody against p63 (clones 463M-17, prediluated, ready to use, Cell Marque Datasheet).

The stained slides were examined without knowing the original histologic diagnosis. As there is no consensual scoring, we evaluated intensity of staining as weak (1+), moderate (2+), and strong (3+), and percentage of staining cells. A case was considered positive when nuclear staining of a single lesional cell or more was found.

### Statistical analysis

The calculation of average age, median age, sex ratio and rate of P63 expression was done using Epi-info software. Sensitivity, specificity, positive predictive value (PPV) and negative predictive value (NPV) were calculated in the GCTOB vs. not GCTOB and P63 positive vs. P63 negative groups using the SPSS software package (version 17).

## Results

The patients’ age ranged between 7 and 59 years with an average of 23.8 years and a median of 21 years. A discrete male predominance was noted (sex ratio = 1.2).

**Figure 4:**
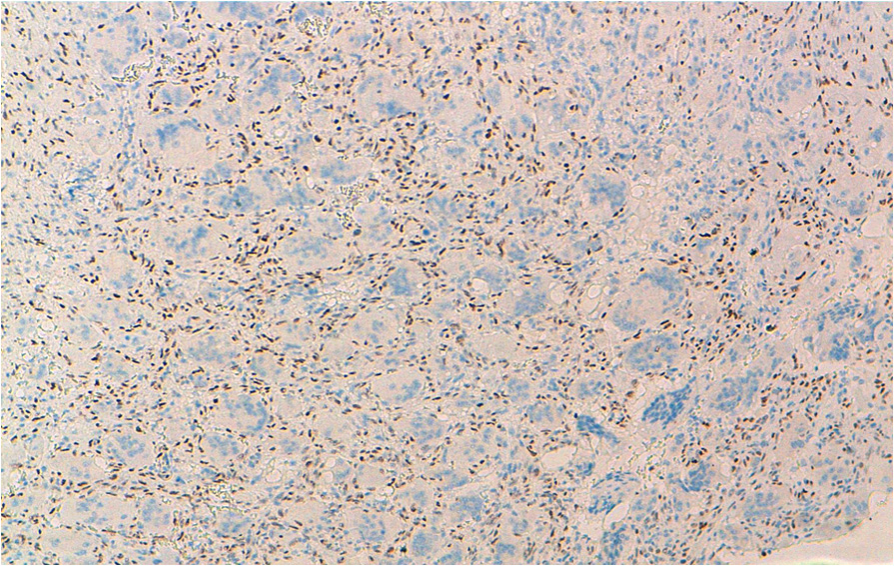
Immunohistochemical findings of GCTOB: strong nuclear staining with P63 in mononuclear cells (original magnification × 100).

**Figure 5:**
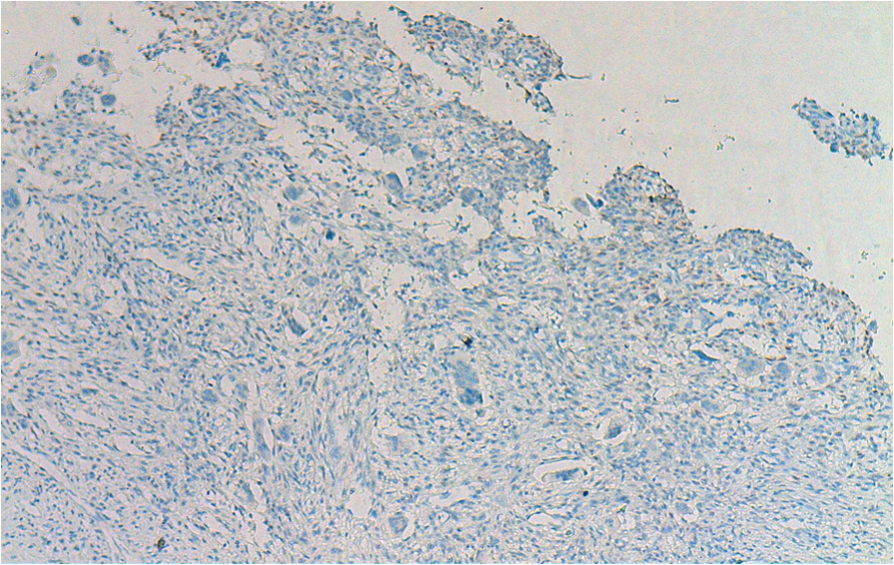
Immunohistochemical findings of ABC: moderate and focal nuclear staining with P63 in mononuclear cells (original magnification × 100).

**Figure 6:**
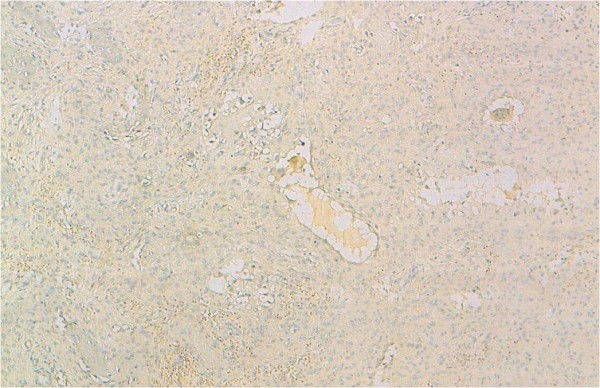
Immunohistochemical findings of CGCG: negative nuclear staining with P63 in mononuclear cells (original magnification × 100).

Immunohistochemical analysis showed a P63 nuclear expression in all GCTOB (Figure 4), 2 of 4 osteoid osteomas (50%), 2 of 5 ABCs (40%) (Figure 5), 3 of 8 osteoblastomas (37.5%), 1 of 3 CMFs (33.3%), 1 of 4 NOFs (25%), 1 of 12 osteosarcomas (8.3%) and in the single case of chondroblastoma included in this series. The staining was observed only in the nucleus of the mononuclear cells and no staining was present in the multinucleated giant cells. No P63 immunoreactivity was detected in any of the cases of CGCG (Figure 6), LCH, and FD. Strong staining was seen in 40% of GCTOB (2 cases) and in one case of osteoblastomas (33.3% of P63 positive osteoblastomas). Moderate staining was seen in 2 cases of GCTOBs (40%) and in one case of ABCs. In other tumors expressing P63, staining intensity was weak. Staining was seen in 30%-60% of tumor cells in GCTOB and in 20% and 50% of tumor cells in ABCs. In other tumors, percentage of reactive cells was lower (5%-30% in osteoblastomas, 10% in osteoid osteomas, osteosarcomas and CMFs, and 5% in chondroblastoma and NOFs).

The sensitivity and negative predictive value (NPV) of P63 immunohistochemistry for the diagnosis of GCTOB were 100%. The specificity and positive predictive value (PPV) were 74.42% and 59.26% respectively.

## Discussion

In this study, we showed that all GCTOBs express P63. Dickson [4] and Linden [15] found similar results by immunohistochemistry. They reported P63 overexpression in all GCTOB. In De la Rosa’s study [3], P63 immunoreactivity was seen in 20 of 23 GCTOBs (86.9%). Similar results were reported by Lee [6] who showed a P63 overexpression by immunohistochemistry in 81% of cases (n=26) with a strong staining in 69% (Table 2). The immunostaining was mostly confined to the mononuclear component [3,4,6]. This strong expression of P63 suggests that this protein may be implicated in the pathogenesis of GCTOB but determining its exact role requires further investigation.

**Table 2:** P63 expression of in current series and in other published series

**Diagnosis**	**Our series**		**Dickson’s series [**[4]**]**		**De la Rosa’s series [**[3]**]**		**Lee’s series [**[6]**]**	
	**N° of cases**	**P63+ cases (%)**	**N° of cases**	**P63+ cases (%)**	**N° of cases**	**P63+ cases (%)**	**N° of cases**	**P63+ cases (%)**
GCTOB	5	100%	17	100%	23	86.9%	6	81%
Osteosarcoma	12	8.3%	0	-	4	50%	13	15.3%
Osteoblastoma	8	37.5%	0	-	0	-	0	-
ABC	5	40%	7	28.6%	8	62.5%	25	20%
CGCG	4	0%	12	0%	4	100%	12	0%
NOF	4	25%	0	-	6	16.6%	0	-
OO	4	50%	0	-	0	-	0	-
CMF	3	33.4%	0	-	0	-	12	0%
Chondroblastoma	1	100%	10	30%	12	83.3%	15	40%
FD	1	0%	0	-	2	0%	4	0%
LCH	1	0%	0	-	0	-	0	-
chondrosarcoma	0	-	0	-	0	-	0	-
Brown tumor	0	-	0	-	0	-	4	0%

The relationship of GCTOB and central giant cell granuloma has long been controversial. The absence of p63 expression in CGCG suggests that these tumors may have a pathogenesis that differs from that of GCTOB. P63 negativity found in all cases of CGCG in our study is consistent with results obtained by Dickson [4] and Lee [6] who found negativity in all cases (n=12 in each series). De la Rosa [3] showed different results with p63 positivity in all cases (n = 4) (Table 2).

Only one case (8.3%) of osteosarcomas included in our study showed overexpression of P63. Proportion of immunoreactive cells was less than 10% and staining was 1+ in intensity. The rate of P63 expression in other series remains low (2 cases/13 in Lee’s study, with low intensity [6], and 2 cases/4 in De la Rosa’s study [3]) (Table 2).

In this work, we recorded a single case of chondroblastoma. The immunohistochemical study showed P63 expression by less than 10% of tumor cells with low intensity. The rate of expression in other studies is variable. In Dickson’s study, 3 of 10 chondroblastomas expressed p63 (30.0%); this ranged from 7–75% of cells, and staining was predominantly mild-moderate in intensity [4]. De Larosa found a higher expression (83.3%, 10 of 12 chondroblastomas) with moderate staining in 6 cases, weak staining in 3 cases and strong staining in only one case [3]. Lee showed P63 staining in 40% of cases (6 of 15). To differentiate between chondroblastoma expressing P63 and GCTOB, he used PS100: chondroblastoma shows positive S-100 immunostaining whereas only occasional weak S-100 immunostaining is seen in GCTOB [6]. In the same study, no P63 staining was seen in chondromyxoïd fibromas (n=12) (Table 2).

The rate of P63 expression in ABC in Dickson’s [4] and Lee’s [6] studies is lower than that obtained in our study: 28.6% (2 cases/7) and 20% (5cases/25) respectively. De la Rosa [3] and Linden [15] found higher results: 62.5% and 100% respectively (Table 2). If some cases of ABC are P63 +, they could be a component of a GCTOB.

In fibrous dysplasia, our results are concordant with those found by De La Rosa [3] (two cases all negative) and Lee (4 cases all negative) [6]. Non ossifiant fibroma showed P63 expression in one case with weak and focal staining. De la Rosa found similar results with P63 expression in 1 of 6 cases (16.6%) (Table 2). Proportion of positive cells was less than 10% and staining intensity was weak [3].

In current study, 50% of osteoid osteomas and 37.5% of osteoblastomas expressed P63. LCH showed no P63 immunostaining. These tumors were not included in the other studies.

P63 contribution in the differential diagnosis between GCTOB and other multinucleated giant cell-containing lesions of bone is variable. Dickson [4] considers that P63 can be useful as a biomarker for the differential diagnosis between GCTOB and other lesions particularly central giant cell granuloma, since these do not express P63. De La Rosa [3] found a high P63 negative predictive value (91.17%) but a low specificity (53.36%) which limits the use of this protein as an immunohistochemical marker for differential diagnosis. Lee [6] considers that the use of P63 can help in histological diagnosis of GCTOB. In current study, the P63 negative predictive value is 100%, this means that in difficult cases, P63 negativity can eliminate a GCTOB. The positive predictive value is low (59.26%). However, except a case of osteoblastoma, a strong staining was found only in GCTOB. Therefore, it is strongly suggestive of this tumor.

## Conclusion

This study shows that P63 may serve as a biomarker for the differential diagnosis between GCTOB and other morphologically similar lesions, particularly CGCG since the latter does not express P63. Other giant cell-containing lesions express P63, decreasing its specificity as a diagnostic marker, but a strong staining was seen, except a case of chondroblastoma, only in GCTOB.

## Abbreviations

ABC: Aneurysmal bone cyst; CGCG: Central giant cell granuloma; CMF: Chondromyxoid fibroma; FD: Fibrous dysplasia; GCTOB: Giant cell tumor of bone; LCH: Langerhans cell histiocytosis; NOF: Non ossifiant fibroma; OO: Osteoid osteomas.

## Competing interests

The authors declare that they have no competing interests.

## Authors’ contributions

NH, LC, and AA performed the histological examination of bone lesions and were major contributors to writing the manuscript. HE and TH assisted in histological interpretation. YA and ST performed the radiological examination. All authors read and approved the final manuscript.
